# SH3KBP1 Promotes Glioblastoma Tumorigenesis by Activating EGFR Signaling

**DOI:** 10.3389/fonc.2020.583984

**Published:** 2021-02-11

**Authors:** Hai Song, Yanpei Wang, Chaojia Shi, Jianxiang Lu, Tian Yuan, Xiangpeng Wang

**Affiliations:** Department of Neurosurgery, First Affiliated Hospital of Kunming Medical University, Kunming, China

**Keywords:** glioblastoma, SH3KBP1, epidermal growth factor receptor, adaptor, glioblastoma stem cells

## Abstract

Glioblastoma (GBM) is the most common and aggressive brain tumor in adults. Overexpression or activation of epidermal growth factor receptor (EGFR) occurs commonly in multiple human cancers and promotes tumorigenesis. However, the underlying molecular mechanism of EGFR aberrant activation and the downstream signaling pathways remains largely unknown. In this study, we report that both SH3-domain kinase binding protein 1 (SH3KBP1) mRNA and protein levels are highly expressed in GBM and its high expression is associated with worse survival of glioma patients. In addition, we provide evidence that SH3KBP1 is prominently expressed in GBM stem cells (GSCs) and have potential to serve as a novel GSCs marker. Moreover, silencing SH3KBP1 dramatically impairs GBM cell proliferation, migration and GSCs self-renewal ability *in vitro* and xenograft tumors growth *in vivo*. Most importantly, we found that SH3KBP1 directly interacts with EGFR and may act as an adaptor protein to transduce EGFR signaling. Together, our work uncovers SH3KBP1 as a novel regulator of oncogenic EGFR signaling and also as a potential therapeutic target for GBM patients with EGFR activation.

## Introduction

Glioblastoma (GBM, WHO IV glioma) is the most common and lethal tumor of the central nervous system, which accounts for 15.4% of all primary brain tumors and 45.6% of primary malignant brain tumors (1–3). Despite recent advances in GBM clinical treatment, the overall survival of GBM patients remains only 15 months (4, 5). Glioma stem cells (GSCs), which are a small subpopulation of cells reside at GBM niches and are characterized by having self-renewal ability and tumor-initiating capacity simultaneously, which have shown to account for GBM initiation, therapeutic resistance and tumor relapse (6–8). Thus, new therapies that specifically delete GSCs may be developed by promising initiatives, which are urgently needed for glioma patients’ clinical therapy.

A hallmark of human cancers is aberrantly active oncogenic signaling activated by amplified and overexpressed oncogenes (9). Epidermal growth factor receptor (EGFR) is a trans-membrane receptor tyrosine kinase of the ERBB family that is an essential enzyme for cell proliferation and tumorigenesis (10). Upon binding to ligands, including EGF or TGF-α, EGFR forms heterodimers or homodimers, leading to its C-terminal tail autophosphorylation and activates downstream signaling through its docking site of SRC homology domain (11). EGFR signaling pathways have well been established to play essential roles in regulating fundamental development stage and pathological processes. An overexpression of either EGFR or EGFR-activating mutations has been documented in various human tumors and is correlated with a poor clinical prognosis (12–14). It is well established that activation of EGFR signaling plays critical roles in promoting multiple tumor cell proliferation, migration, therapeutic resistance and cancer stem cell self-renewal maintenance (15, 16). Thus, it is important to identify more therapies targeting EGFR as new clinically applicable drugs.

SRC homology 3 (SH3) domain is composed of 50 amino acid residues, which is an evolutionarily conserved sequence in the non-catalytic part of receptor tyrosine kinases (17). SH3KBP1 was functionally identified as an adaptor protein and plays key roles in activating B cells and preventing EGFR degradation *via* its two PXXXPR motifs in carboxyl terminus, which specifically binds to SH3 domain kinases (18, 19). In cancer initiation and development, SH3KBP1 has been reported to inhibit Prolyl Hydroxylase 2 (PHD2) binding with Hypoxia-Inducible Factor (HIF) and promotes cancer progression (20). In addition, SH3KBP1 has been documented to interact with c-Cbl and promote breast cancer cell invasion (21). However, the biological function of SH3KBP1 and its underlying molecular mechanism in regulating glioma tumorigenesis remains largely unknown. Based on this background, we focused on investigating the functional roles of SH3KBP1 in glioma tumorigenesis.

## Materials and Methods

### Tissues Collection

Total of 27 pairs of glioma samples were collected from First Affiliated Hospital of Kunming Medical University and the study was carried out under the approval of Ethics Committee of First Affiliated Hospital of Kunming Medical University. Patients who received either radiotherapy or chemotherapy before surgery were excluded. Prior to this study, all patients signed the written informed consent.

### Cell Lines and Transfection

Human GBM cell lines (U87, U251, and LN229) used in this study were all purchased from ATCC (Manassas, USA) and then cultured in DMEM (Life Technologies). The DMEM culture medium was supplement with 10% FBS and 1% antibiotics. Cells were all cultured under a chamber with 95% O_2_, 5% CO_2_ and 37°C.

For lentiviral short hairpin RNA (shRNA) transfection, control shRNA (Ctrl shRNA) or target SH3KBP1 shRNA were cloned into pLKO.1 and co-transfected into 293T with pMD2.G and pSPAX2, respectively. After 48 h, the virus particles were harvested and passed through a 0.22-mm filter. The parental cells were infected with the virus supernatant for another 48 h and selected using 1 µg/ml puromycin for 7 days. After selection, the stable cell lines were used for further study. Sequence of shRNA was as follow. SH3KBP1 shRNA: CCGCAGACTTGTCTGAGATTG. Control shRNA: GCAGCCTAACGACATGAT.

### Immunofluorescence Staining

Briefly, 4% paraformaldehyde was used to fix human surgical glioma specimens for 15 min. Samples were blocked with 10% normal donkey serum with 0.2% Triton X-100 in PBS for 60 min at room temperature and then incubated with primary antibodies overnight at 4°C, followed by incubation with appropriate secondary fluorescently labeled antibodies (Invitrogen) for 2 h at room temperature. Nuclei were counterstained with Hoechst. Two GBM specimens were used in this study.

### Immunohistochemistry (IHC)

Human tumor or mouse xenograft tumor tissues were fixed in 4% paraformaldehyde, dehydrated and embedded in paraffin. Then, paraffin-embedded tissues were cut into 4-µm thick sections. After being de-paraffinized and rehydrated through a descending alcohol series, the sections were followed by antigen retrieval. After blocking with 1%BSA for 30 min at room temperature, the sections were incubated with antibodies against Ki67 and p-STAT3 overnight at 4°C and incubate with secondary antibody conjugates with HRP for 30 min at 37°C and examined by using diaminobenzidine detection. Thereafter, sections were stained with hematoxylin for nuclear staining and finally observed and photographed under a microscope. SH3KBP1 (1:100) (Cell signaling technology, 12304), Ki67 (1:500) (Cell signaling technology, 9449).

### *In Vitro* Limiting Dilution and Spheres Formation Assay

For spheres formation assay, 50,000 U87 and U251 cells expressing with or without SH3KBP1 shRNA were seeded in ultra-low attachment six-well plates (Corning, USA) and cultured in Dulbecco’s modified Eagle’s medium/F12 (Life Technologies) supplemented with B27 (Invitrogen, USA), N2 (Invitrogen, USA), 20 ng/ml epidermal growth factor (Millipore) and 20 ng/ml basic fibroblast growth factor (Millipore). After two weeks, spheres larger than 50 µm were counted and representative images were pictured under stereomicroscope (Olympus, Tokyo, Japan). For *in vitro* limiting dilution assay, single dissociated U87 and U251cells were seeded in ultra-low attachment 96-well plates (Corning, USA) at density of 5, 10, 20, 50, 100, or 200 cells per well with above mentioned sphere formation medium. After culturing without serum for 14 days, formatted spheres were counted in each well and sphere formation frequency was calculated using online *in vitro* limiting dilution analysis (http://bioinf.wehi.edu.au/software/elda/).

### Colony Formation Assay

The single U251 and U87 cells expressing with or without SH3KBP1 shRNA were plated to six-well plates at a density of 3,000 cells per well. Cells were cultured for 14 days to form colony and were fixated with 4% formaldehyde and then stained with 0.5% crystal violet. Colonies visible to the naked eye were imaged and counted manually.

### Wound Healing Assay

Cells were seeded to six-well plates at a density of 600,000 cells per well and the scratch was created by a 200 µl pipette tip when cell grown to 80% confluence. The wound closure was imaged under microscope at 0 h and at 48 h after being maintained in serum-free medium.

### Trans-Well Assay

The 8-µm pore trans-well chamber (Corning, USA) was used for trans-well assay. Briefly, a total of 1 × 10^4^ U251 and U87 cells expressing with or without SH3KBP1 shRNA cells in serum-free medium were plated to the upper chamber. Simultaneously, DMEM culture medium with 20% FBS was added to the lower chamber. After culturing for 24 h, cells which invaded the lower chamber were fixated and stained with 1% crystal violet. Five random fields were pictured and migrated cells were counted.

### EdU Assay

Proliferation ratio of the GBM cell lines was examined by using a Click-iTEdU Kit (Solarbio, CA1170) according to the manufacturer’s protocol. Briefly, 10 μM EdU was added into cells for 2 h before fixation and permeabilization. Cell nuclei were counterstained with Hoechst (Solarbio, C0021) at a concentration of 5 µg/ml for 30 min.

### Quantitative Real Time PCR

Complementary DNA (cDNA) was obtained *via* reverse transcription of messenger RNA (mRNA), and cDNA were used as a template for quantitative real time PCR. Quantitative PCR kit was purchased from TIANGEN BioMart (Beijing, China). We detected mRNA expression level by quantitative PCR with ABI 7500 (Applied Biosystems). GAPDH was used as internal control. Sequences of PCR primers used in this study are shown as below. SH3KBP1 Forward primer: 5’-CATCGACGTAGGCTGGTGG-3’. SH3KBP1 Reverse primer: 5’-CCTTCCTTTTCAAAGTCCGGTG-3’. GAPDH Forward: 5’-GGAGCGAGATCCCTCCAAAAT-3’. GAPDH Reverse: 5’-GGCTGTTGTCATACTTCTCATGG-3’.

### Western Blot

For western blot assay, cells were harvested and lysed with RIPA buffer and protease (Roche) and phosphatase inhibitors (Roche) were added into the RIPA buffer. After boiling for 5 min, the same quality of protein was loaded to SDS–PAGE gels and immunoblotting. GAPDH was used as loading control. After transferring to polyvinylidene fluoride membranes and blocking with 5% milk for 1 h at room temperature, the membranes were incubated with primary antibodies at 4°C for overnight, then incubated with HRP conjugated secondary antibodies. Antibodies used in this study were as shown below; SH3KBP1 (1:500) (12304), STAT3 (1:1,000) (9139), Phospho-STAT3 (1:500) (Tyr705) (9145), EGFR (1:1,000) (4267), AKT (1:1000) (9272), Phospho-AKT (Ser473) (1:500) (4060), MAPK (1:1,000) (9212) and Phospho-MAPK (Tyr180) (1:500) (9211) all purchased from cell signaling technology.

### Co-Immunoprecipitation

Cells were harvested and lysed with RIPA buffer containing protease inhibitors for 30 min at 4°C. Cell lysates were precleared by protein A/G beads (Santa Cruz Biotechnology) for 1 h incubation at 4°C, and precleared protein A/G beads were removed then primary antibody was added for overnight incubation at 4°C, and new protein A/G beads were added for 2 h incubation, then beads were collected by washing with ice cold PBS for four times. Finally, the bound protein was eluted by boiling for 10 min and subjected to SDS–PAGE and detected by immunoblotting. SH3KBP1 (1:500) (Cell signaling technology, 12304), EGFR (1:1,000) (Cell signaling technology, 4267).

### *In Vivo* Tumor Growth Model

For intracranial tumor formation assay, Female NOD/SCID mice of 4–6 weeks were purchased from Shi Laike Company (Shanghai, China). 50,000 U87 cells were intracranially transplanted into the right cerebral cortex (coordinates: 2 mm anterior, 2 mm lateral, 3.5 mm depth from the dura). Mice were sacrificed when exhibiting neurological signs. For xenograft tumor formation assay, 4–6 weeks-old female BALB/c nude mice purchased from Shi Laike Company (Shanghai, China). Xenograft tumor model was created through subcutaneously injection with 1 × 10^6^ U87 cells expressed with control shRNA or SH3KBP1 shRNA to BALB/c nude mice. Tumor volume was recorded twice a week. Six weeks later, mice were sacrificed and tumors derived from indicated cells were collected, weighed and pictured. All animal studies were approved by the Animal Ethics Committee of Kunming Medical University.

### Bioinformatic Analysis

To comprehensively explore the expression pattern and prognostic implications of SH3KBP1 in gliomas, transcriptome sequencing and corresponding clinical data of TCGA, CGGA and Rembrandt data sets were downloaded from http://gliovis.bioinfo.cnio.es/.

### Statistical Analyses

Each assay was repeated independently for three times and experimental data were processed by Prism 7.0 software (GraphPad Software) and shown as mean ± S.E.M. The differences between groups were analyzed *via* Student’s t-test or one-way ANOVA and data were thought significantly different when P-value below 0.05. *, ** and *** indicate as p<0.05, p<0.005 and p<0.001 respectively.

## Results

### Expression and Clinical Implications of SH3KBP1 in Glioma

**Figure 1:**
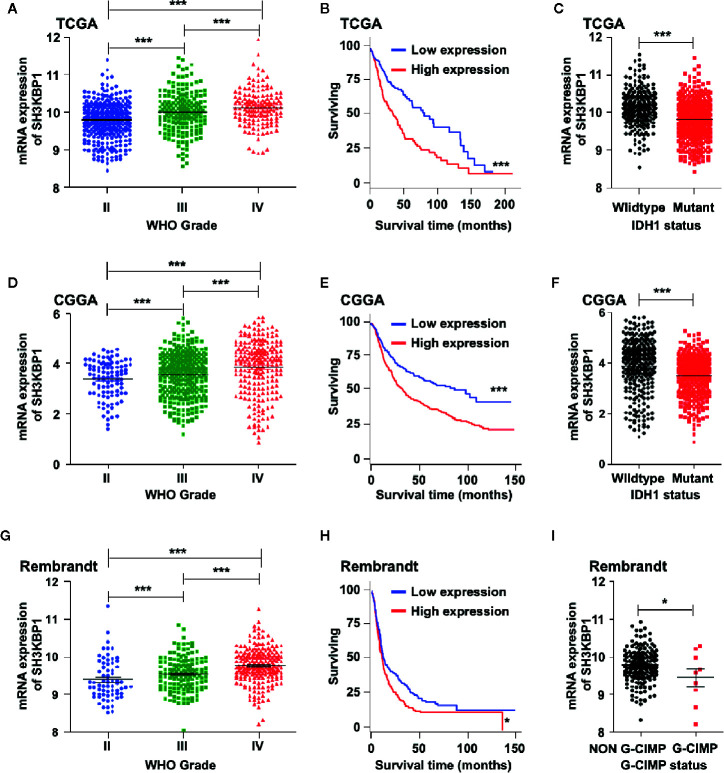
SH3KBP1 is highly expressed in GBM. **(A)** The expression of SH3KBP1 in glioma was increased with the tumor grade in TCGA data set. **(B)** High expression of SH3KBP1 predicts worse survival of glioma patients in TCGA data set. **(C)** SH3KBP1 expression was highly expressed in Isocitrate dehydrogenase1 (IDH1) wild-type gliomas than mutant gliomas in TCGA data set. **(D)** SH3KBP1 expression was correlated with glioma grades in CGGA datasets. **(E)** Elevated expression of SH3KBP1 predicts poor survival of glioma patients in CGGA data set. **(F)** SH3KBP1 was highly expressed in IDH1 wild-type gliomas than mutant gliomas in CGGA data set. **(G)** The expression of SH3KBP1 in glioma was increased with the tumor grades in Rembrandt data set. **(H)** High expression of SH3KBP1 predicts unfavorable survival of glioma patients in Rembrandt data set. **(I)** SH3KBP1 was highly expressed in NON G-CIMP than G-CIMP gliomas in Rembrandt data set. Lines show mean and S.E.M. Log-rank (Mantel-Cox) test was used to analysis the difference of Kaplan-Meier survival analysis. *, ** and *** indicate as p < 0.05, p < 0.005 and p < 0.001 respectively.

To identify the potential biological functions of SH3KBP1 in glioma initiation and progression, we assessed the SH3KBP1expression pattern in different grades of gliomas by using public datasets TCGA (22) and observed that SH3KBP1 expression positively correlates with tumor grades of glioma patients (**Figure 1A**). In addition, we performed Kaplan-Meier survival analysis in TCGA data set and the results showed a statistically significant worse prognosis for glioma patients with higher expression of SH3KBP1 compared with lower SH3KBP1 group patients (**Figure 1B**). It has been well recognized that patients with Isocitrate dehydrogenase1 (IDH1) mutant gliomas frequently have a favorable prognosis compared with wild-type (WT) IDH tumors’ patients (23). It is worth noting that, by comparing the expression of SH3KBP1 in IDH1 wild-type and mutant groups, we observed that SH3KBP1 was highly expressed in wild-type IDH1 tumors, implying that SH3KBP1 may play critical roles in glioma progression (**Figure 1C**). Consistently, we observed almost exactly the same patterns in CGGA (**Figures 1D–F**) and Rembrandt glioma datasets (**Figures 1G, H**). Glioma CpG island methylator phenotype (G-CIMP) is another widely accepted prognosis marker of gliomas and glioma patients with NON G-CIMP always predict poor prognosis, thus we compared the expression levels of SH3KBP1 in G-CIMP and NON G-CIMP group and found that SH3KBP1 was highly expressed in NON G-CIMP group (**Figure 1I**). Together, these data revealed that the expression level of SH3KBP1 is an indicator of the aggressiveness of malignant gliomas and SH3KBP1 could be used as a biomarker for glioma prognosis.

### SH3KBP1 Is Elevated in GSCs

**Figure 2:**
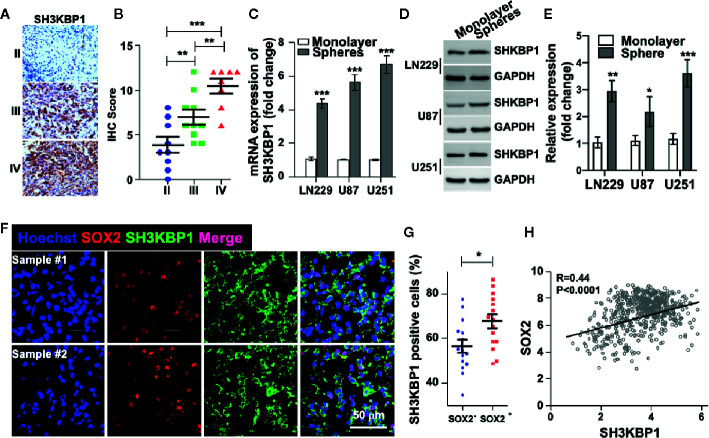
SH3KBP1 is elevated in glioma stem cells (GSCs). **(A)** Representative images of immunohistochemistry (IHC) staining of SH3KBP1 from 27 gliomas specimens. **(B)** Quantitative data for **(A)**. **(C)** Real-time PCR analysis showing the expression of SH3KBP1 in tumor spheres and their corresponding monolayer indicated cells. **(D)** Western blot assay was performed to analyze the expression pattern of SH3KBP1 in tumor spheres and their corresponding monolayer indicated cells. **(E)** Quantification data for **(D)**. **(F)** Representative images of immunofluorescence staining of SH3KBP1 and SOX2 in two independent Glioblastoma (GBM) specimens. **(G)** Five randomly selected microscope fields of each tumor were quantified. **(H)** Pearson correlation between SH3KBP1 and SOX2 mRNA expression in the CGGA Glioma dataset. Pearson correlation coefficients are shown in the matrix. *, ** and *** indicate as p < 0.05, p < 0.005 and p < 0.001 respectively.

In order to examine the protein levels of SH3KBP1 *in vivo*, we performed immunohistochemistry (IHC) staining using 27 clinical glioma patients’ specimens, including nine WHO grade II, 10 WHO grade III, eight WHO grade IV. As shown in (**Figure 2A** and **Table 1**), SH3KBP1 staining was weak or negative in WHO grade II tumors. SH3KBP1 was expressed in majority of high-grade gliomas, particularly in GBM specimens. The quantification results indicated a strong positive association between the expression level of SH3KBP1 and glioma grade (**Figure 2B**).

**Table 1:** Clinicopathological characteristics of patients with glioma.

Patients characteristics	No. of patients (%)
**glioma**	
**Age(years)**	
≤50	12(44.5)
>50	15(55.4)
**Gender**	
Male	17(63.0)
Female	10(37.0)
**Clinical stages**	
Stage II	9(33.3)
Stage III	10(37)
Stage IV	8(30.0)

Cancer stem cell (CSC) model suggests that a subpopulation cell residing in tumor is responsible for cancer initiation, therapeutic resistance and tumor relapse (6–8, 24). Thus, we wondered whether SH3KBP1 also plays essential roles in glioma stem cell (GSC). To test this hypothesis, we performed tumor spheres formation assay to enrich GSCs. Interestingly, we found that both SH3KBP1 mRNA and protein expression levels were dramatically increased in tumor spheres derived from U251, U87, and LN229 cells than their corresponding monolayer cells (**Figures 2C–E**), suggesting that SH3KBP1 plays critical roles in regulating GSCs self-renewal. Next, we validated this observation by performing immunofluorescence analysis in clinical GBM patients’ specimens and observed that SH3KBP1 are markedly co-expressed with SOX2, which is a known GSCs marker (**Figures 2F, G**). Consistent with this finding, we found that SH3KBP1 mRNA expression positively correlated with SOX2 mRNA expression in TCGA data set (**Figure 2H**) and CD133 and Oct4 mRNA (data not shown), which is also another widely recognized GSCs marker. Collectively, these data showed that SH3KBP1 is highly expressed in GBM and GSCs, which might be used as a new GSCs marker.

### SH3KBP1 Promotes GBM Cell Proliferation and Migration

**Figure 3:**
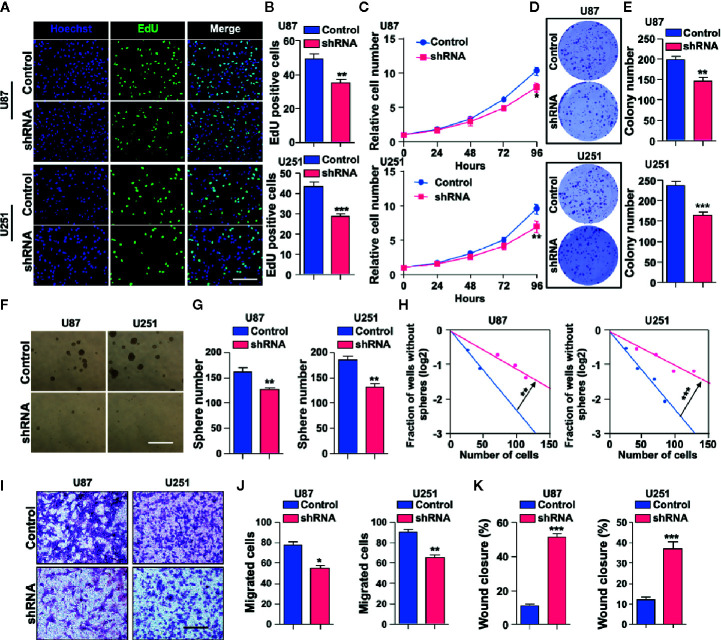
Depletion of SH3KBP1 significantly inhibits Glioblastoma (GBM) cell malignant behaviors. **(A)** Indicated cells’ proliferation rate was significantly decreased following SH3KBP1 silencing determined by EdU incorporation assay. Scale bar: 200 µm. **(B)** Quantitative data for **(A)**. **(C)** GBM cell proliferation was dramatically inhibited by knocking down SH3KBP1 as determined by cell proliferation assay. **(D)** Colony formation ability of indicated cells was significantly suppressed by silencing SH3KBP1. **(E)** Quantitative data for **(D)**. **(F)** Depletion SH3KBP1 significantly inhibits indicated cells spheres formation ability. Scale bar: 500 µm. **(G)** Quantitative data for **(F)**. **(H)** Tumor sphere formation ability was significantly inhibited following SH3KBP1 silencing determined by *in vitro* limiting dilution assay. **(I)** GBM cell migration ability was significantly inhibited by SH3KBP1 depletion in the indicated cells. **(J)** Quantitative data for panel **H**. **(K)** GBM cell migration ability was significantly inhibited by depletion SH3KBP1 in indicated cells determined by wound healing assay and the quantitative data have shown. *, ** and *** indicate as p < 0.05, p < 0.005 and p < 0.001 respectively.

To further study the biological function of SH3KBP1 in GBM development and progression, we used short hairpin RNA (shRNA)-expressing plasmids to generate stable SH3KBP1 knockdown clones in U251 and U87 cells. Silencing SH3KBP1 impeded U251 and U87 cell growth determined by EdU incorporation assay (**Figures 3A, B**). This finding was further confirmed by cell proliferation results. As shown in (**Figure 3C**), U251 and U87 SH3KBP1 silencing cells proliferation ratio was suppressed compared with its corresponding control cells. Consistently, colony formation ability was significantly inhibited following silencing of SH3KBP1 in U251 and U87 cells (**Figures 3D, E**). We then examined anchorage-independent sphere formation and found that compared to the control cells, SH3KBP1 knockdown cells exhibited significantly weaker tumor sphere-tumor formation ability in U251 and U87 (**Figures 3F, G**). This observation was further validated by performing an *in vitro* limiting dilution assay (**Figure 3H**), which revealed that tumor spheres formation ability was significantly inhibited following SH3KBP1 silencing, suggesting that SH3KBP1 play an essential role in regulating GSCs self-renewal abilities.

Tumor dispersal is a critical problem in the treatment of brain tumors and invading cells are highly resistant to radiation and chemotherapy, which is a culprit of recurrence (25–27). To explore the functional roles of SH3KBP1 in GBM migration, trans-well and wound healing assay were performed. As shown in (**Figures 3I–K**), U251 and U87 cells’ migration ability was significantly inhibited by silencing SH3KBP1. Collectively, these data indicate that SH3KBP1 is essential for GBM cell proliferation and migration as well as GSCs self-renewal.

### SH3KBP1 Directly Interacts With and Activates EGFR

**Figure 4:**
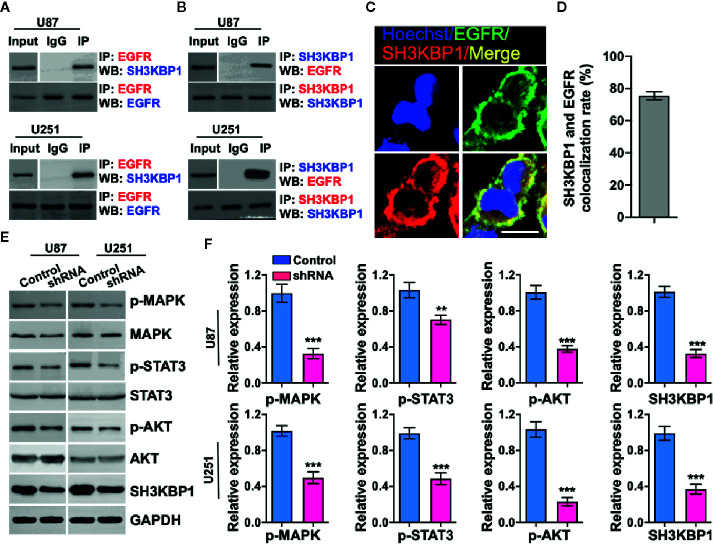
SH3KBP1 directly and physically interacts with and activates epidermal growth factor receptor (EGFR) signaling. **(A)** SH3KBP1 physically interacts with EGFR determined by Co-IP assay. **(B)** EGFR physically interacts with SH3KBP1 determined by Co-IP assay. **(C)** Confocal analysis of the co-localization of SH3KBP1 and EGFR in U87 cells. Cells were stained with SH3KBP1 (red), EGFR antibody (green) and merged images are shown. Scale bar: 50 µm. **(D)** Quantitative data for **(C)** (n=6). **(E)** Depletion of SH3KBP1 significantly inhibits EGFR signaling downstream genes expression. **(F)** Quantitative data for **(E)**. *, ** and *** indicate as p < 0.05, p < 0.005 and p < 0.001 respectively.

Given that adaptor protein is involved in regulating diverse signal transduction pathways, we speculated that biological function of SH3KBP1 may be dependent on binding with proteins having SRC homology domain. Thus, we examined whether SH3KBP1 directly interacts with EGFR, which is a well-known kinase with a SRC homology domain. We performed endogenous immunoprecipitation and western blot analysis and observed that SH3KBP1 binds to EGFR (**Figure 4A**), and EGFR also binds directly to SH3KBP1 (**Figure 4B**). Consistently, immunofluorescence also manifested the co-localization of SH3KBP1 with some endogenous EGFR on cell membrane of U87 cells (**Figures 4C, D**). In addition, we found that p-AKT, p-MAPK and p-STAT3, which are key downstream regulators of EGFR signaling pathways, were all simultaneously decreased after depletion of SH3KBP1 (**Figures 4E, F**), suggesting that SH3KBP1 may acts as an adaptor protein that plays important roles in EGFR signaling transduction.

### SH3KBP1 Enhances GBM Progression *In Vivo*

**Figure 5:**
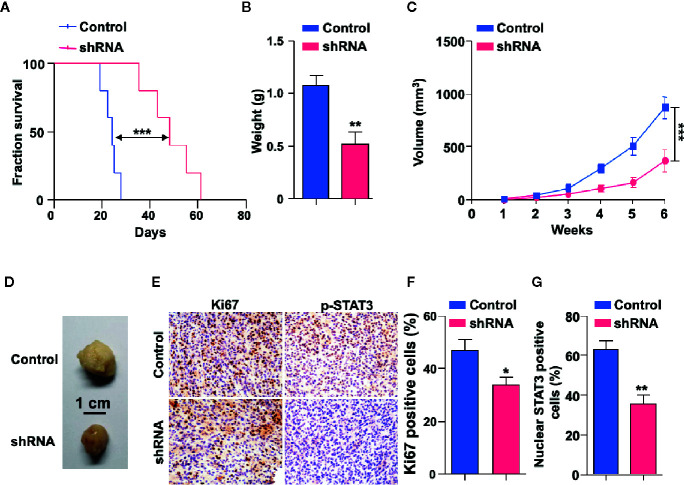
Depletion of SH3KBP1 inhibits mouse xenograft tumor growth *in vivo*. **(A)** Kaplan-Meier survival analysis of mice intracranially implanted with U87 cells with or without SH3KBP1 knockdown. n = 5, log-rank (Mantel-Cox) test. **(B)** Tumor weight was measured in mice after the mouse was sacrificed. **(C)** SH3KBP1 knockdown significantly inhibited mouse xenograft tumor growth. **(D)** Representative tumors derived from indicated cells were imaged. n = 5. **(E)** Immunohistochemistry staining analysis of Ki67, and p-STAT3 in U87 cells derived mouse xenograft tumors. Five tumors from each group were analyzed. **(F, G)** Quantitative data for **(E)**. *, ** and *** indicate as p < 0.05, p < 0.005 and p < 0.001 respectively.

To explore the potential functions of SH3KBP1 in GBM progression *in vivo*, we utilized an intracranially transplanted tumor xenograft model in NOD/SCID mice with U87 cells. We injected 50,000 U87 control or SH3KBP1 silencing cells into the brain of NOD/SCID nude mice and observed that those injected with SH3KBP1 shRNA-U87 cells displayed longer survival (**Figure 5A**). Xenograft tumor model were further performed to verify this observation and found that SH3KBP1 knockdown significantly inhibited tumor growth (**Figures 5C, D**) and tumor weight in xenograft mouse tumors (**Figure 5B**). Most importantly, immunohistochemistry assay was performed on tumors derived from mouse xenograft and found that SH3KBP1 silencing tumors exhibited a dramatic decrease in Ki67, a marker of proliferating cells, positive cells and p-STAT3 (Y705) than control tumors sections (**Figures 5E–G**). These data indicated that depletion of SH3KBP1 inhibits GBM tumorigenesis *in vivo*.

## Discussion

Glioblastoma is one of the most common and aggressive tumors in adults (2, 3). GBM is not a surgically curable disease as tumor cells invade the surrounding brain, which renders a completely impossible surgical resection. Furthermore, GBM tumors are among the most resistant tumors to radiation and cytotoxic chemotherapy (28, 29). Currently, survival time of GBM patients remains only at 15 months despite aggressive surgical resection and conventional therapy (30). Thus, a deeper understanding of molecular mechanism involved in GBM initiation and development is essential for development of more effective therapies for this lethal disease.

Numerous studies have reported that EGFR activation promotes multiple tumor progression (14, 31). Activation of EGFR by binding with ligand or mutation is essential for tumor cell proliferation, migration and cancer stem cell self-renewal. GBM has been characterized by amplification or activation of EGFR (32, 33). EGFR drives tumorigenesis by multiple down-stream signaling pathways thereby stimulating cancer cell proliferation, survival and chemoresistance (34). In this study, we report a novel function of SH3KBP1, acting as a new downstream effector of EGFR signaling to mediate EGFR-driven tumorigenesis. We demonstrate that both mRNA and protein levels of SH3KBP1 are highly expressed in GBM (WHO IV) patients than gliomas (WHO II and III) patients and its high expression predicts poor survival of glioma patients. Furthermore, SH3KBP1 was also upregulated in tumor spheres than monolayer cells and plays critical roles in GSCs self-renewal and stemness maintenance. The importance of this high expression is highlighted by the co-expression of SH3KBP1 and SOX2 in glioma clinical samples. Hodonsky et al. reported that SOX10 binds to SH3KBP1 promoter and regulates SH3KBP1 expression in a transcriptional level (35). Additionally, SOX10 have been identified to play essential roles in cancer stem cell self-renewal (36, 37). Thus, the high expression of SH3KBP1 in GBM may be attributed to SOX10 high expression in GBM and the mechanisms mediating SH3KBP1 overexpression in glioma and GSCs needs further characterized.

Recently, Yao et al. found that EGFR blockade prevents glioma escape from BRAFV600E targeted therapy (38). EGFR inhibitor significantly enhances cisplatin sensitivity of human glioma cells (39). GATA2, hematopoietic factor and gain of function assay revealed that GATA2 significantly enhanced proliferation, migration and invasion of glioma cells by activating EGFR signaling. Glioma patients accepted EGFR-targeted therapy have favorable clinical outcomes (40). However, EGFR mutations in GBM are fully virtually clustered in the extracellular domain and cause the receptor to adopt an inactive conformation. Pharmacological factors and EGFR inhibitors which did not achieve favorable clinical outcomes was caused by the inactive conformation. Thus, there is an urgent need to identify new agents targeting EGFR downstream genes or adaptor proteins as clinically applicable targets to develop to clinical therapy. Sang et al. identified TRIM59 as a new regulator of oncogenic EGFR signaling and silencing TRIM59 inhibits gliomagenesis by inhibiting dephosphorylation of STAT3 (41). Here, we demonstrate that SH3KBP1 interacts physically with EGFR, thereby promoting EGFR activation and glioma tumorigenesis *in vitro* and *in vivo*. However, a detailed molecular mechanism of EGFR signaling activation by SH3KBP1 should be further investigated. Taken together, our study provides clinical and mechanistic evidence demonstrating that SH3KBP1 high expression is essential for EGFR driven tumorigenesis in glioma.

**Figure 6:**
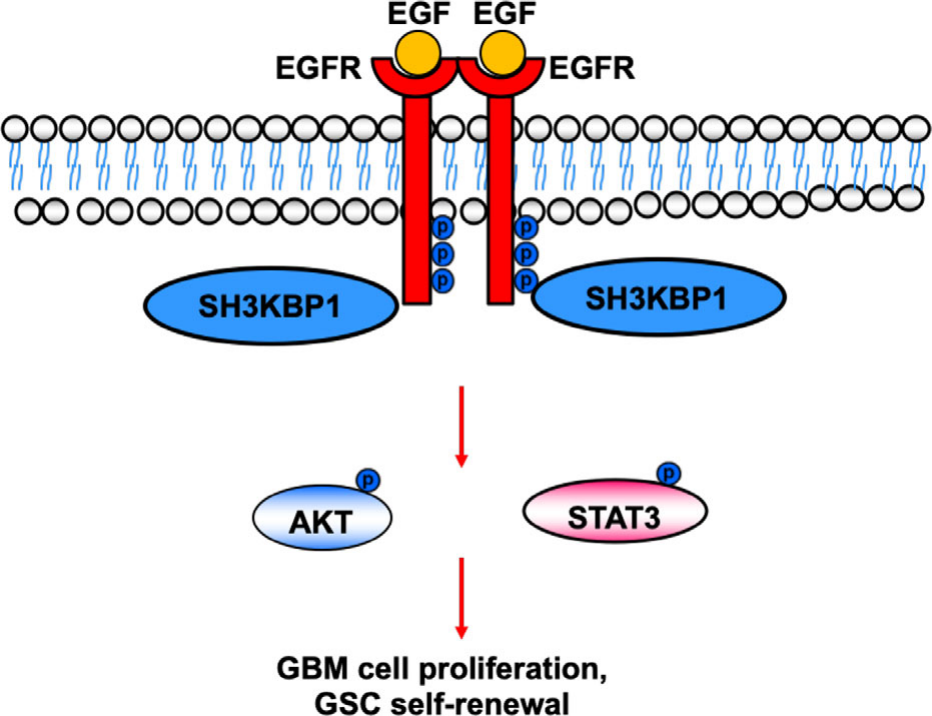
Working model. SH3KBP1 physically interacts with and transduce epidermal growth factor receptor (EGFR) signaling to facilitate Glioma stem cells (GSC self-renewal and GBM cell proliferation.

In this study, we provide evidence that silencing SH3KBP1 significantly inhibits GBM cells proliferation, migration and GSCs spheres formation by functioning as adaptor protein transduces the EGFR signaling. Additionally, targeting SH3KBP1 markedly inhibits EGFR downstream signaling, including p-AKT, p-ERK and p-MAPK expression level (**Figure 6**). Consistently, Feng et al. found that SH3KBP1 prevents epidermal growth factor receptor degradation and activates EGFR signaling (19). EGFR is a well-accepted kinase that plays key roles in glioma cell proliferation and migration and SH3KBP1 activates EGFR signaling. Thus, we concluded that SH3KBP1 promotes glioma progression dependent on SH3KBP1-EGFR axis (**Figure 6**). Inhibitors or drugs that specifically decrease the expression of SH3KBP1 in glioma cells may be developed to increase the lifespan of glioma patients. In the near future, we will focus on screening those inhibitors specifically to determine their inhibitory effect on EGFR/SH3KBP1 signaling. This study therefore presents evidence showing that SH3KBP1 not only can be used as a diagnostic marker but also as a target for therapy in future.

In conclusion, our findings reveal a previously unknown signal relay by which SH3KBP1 mediates EGFR activation, thereby enhancing the oncogenic activity of the EGFR signaling pathway in human gliomas. The newly established roles of SH3KBP1 in EGFR-driven tumorigenesis provide a rationale for SH3KBP1 as a novel prognostic marker for patients with glioma and a potential target for further therapeutic investigation.

## Data Availability Statement

The raw data supporting the conclusions of this article will be made available by the authors, without undue reservation.

## Ethics Statement

The studies involving human participants were reviewed and approved by the Ethics Committee of First Affiliated Hospital of Kunming Medical University. The patients provided their written informed consent to participate in this study.

## Author Contributions

XW designed the study, analyzed data and wrote the manuscript. HS performed almost all experiments. YW performed database analysis. CS, TY, and JL collected GBM clinical samples and performed IHC assay. All authors contributed to the article and approved the submitted version.

## Funding

This study was supported in part by Grants from (1) Health Commission of Yunnan Province Training plan for Medical Reserve Talents (H-2017029. (2) Yunnan Health Science and Technology Project (internal organization) (2017NS058). (3) Research Innovation team of Yunnan province (2019HC022). (4) Yunnan clinical medical center of nervous system diseases (ZX2019-03-05).

## Conflict of Interest

The authors declare that the research was conducted in the absence of any commercial or financial relationships that could be construed as a potential conflict of interest.
